# Association between 25-Hydroxy Vitamin D and volumetric breast density via a fully automated software Volpara™ in the reproductive age group

**DOI:** 10.12669/pjms.325.10731

**Published:** 2016

**Authors:** Bushra Wasim, Khalid Khan, Mohd Abdul Samad

**Affiliations:** 1 Dr. Bushra Wasim, MBBS, FCPS. Professor of Anatomy, Ziauddin University, Clifton Campus, Karachi, Pakistan; 2 Dr. Khalid M. Khan, PhD. Professor, Department of Anatomy, Faculty of Medicine, Kuwait University, Kuwait; 3 Dr. Muhammad Abdul Samad, Msc. International Health (Aberdeen) Research Associate, Shaheen Research Group, Karachi, Pakistan

**Keywords:** 25(OH)D, Breast Cancer, breast density, mammographic density, Volpara, premenopausal

## Abstract

**Objective:**

To determine the association between serum 25 hydroxyvitamin D levels and percent breast density among asymptomatic premenopausal women.

**Methods:**

Hundred asymptomatic, pre-menopausal women who visited the General Surgery Breast Clinic, Patel Hospital, Karachi, Pakistan between 3rd March and 10th November, 2015 were included in this study. The serum 25 (OH)D and calcium levels were measured and mammographic density (MD) was assessed using automated volumetric breast density software, Volpara Research (algorithm version 1.5.1, Volpara solutions Ltd, Wellington, NZ) on the same day. The volumetric breast density (VBD) was categorized as; VG1: 0% - 4.5 %; VG2: 4.6% - 7.5%; VG3: 7.6% – 15.5% and VG4 >15.5%. Mean serum 25(OH)D and calcium levels were compared across the four volumetric breast density categories. The percent volumetric density was also correlated with anthropometric measurements and other related variables.

**Results:**

No significant difference was found in mean serum 25 (OH)D level across the four groups (15.87 Vs. 12.40 Vs. 8.99 Vs. 9.68; p-value = 0.106). The percent VBD were found significantly negatively correlated with age (r = - 0.365; p-value = 0.001), weight (r = - 0.575; p-value = 0.001), height (r = - 0.197; p-value = 0.049), and BMI (r = - 0.519; p-value = 0.001). The serum Vitamin D, and calcium levels were not found significantly correlated with percent VBD (p-value > 0.05).

**Conclusion:**

No significant association exists between serum 25(OH)D level and breast density.

## INTRODUCTION

Breast cancer is one of the most prevalent cancers worldwide,^1^ with more than 1.2 million people being diagnosed with the disease yearly.^2^ The incidence of breast cancer has been reported highest among Jewish women and Asian population.^3^ Breast cancer incidence is also on the rise in Pakistan, with one in every nine females being affected.^4^ It is the most frequent cancer reported among women in Karachi, accounting for one third of the cancers in females.^4^ Despite, the cultural norms of Pakistani women where early marriages, multiparty and longer breast feeding are common, Pakistani women are still at high risk of developing breast cancer.

Recently, there has been much focus on modifiable risk factors i.e. mammographic density (MD), obesity and dietary factors. MD refers to the relative proportions of radiolucent fat and radio dense fibro glandular tissue within the breast on mammography,^5^ and has been identified as an independent and significant risk factor for developing breast cancer. It has been reported that the risk of breast cancer in women with the highest MD is four to six times higher.^1^ Given the consistency of evidence and the strong magnitude of the associations observed, mammographic density may be an intermediate biomarker of breast cancer and has been used as a surrogate endpoint for breast cancer risk in some studies.^6^ MD is typically assessed using the American College of Radiology’s BI-RADS (Breast Imaging-Reporting and Data System) breast composition categories.^7^ However, due to the subjective, visual nature of BI-RADS, automated quantitative methods have now been developed.

Vitamin D, which influences cell proliferation, calcium absorption and breast tissue characteristics have been found inversely correlated with the risk of breast cancer. The results of the meta-analysis reported an inverse association between vitamin D intake and the risk of breast cancer in both pre and post-menopausal women.^8^ Another study conducted in United States in 2006 among 487 women reported no significant association between serum vitamin D levels and breast density, however percent breast density was lowest among those in the highest vitamin D quartile.^9^ The study had a time lag of up to 8 years between blood sampling and mammography. Thus, the possibility remains of the confounding effect by time lag between serum vitamin D measurements and mammography.

The results of previous studies identifying an association between vitamin D and breast density were inconsistent. Thus, this study was conducted to evaluate the association between vitamin D and breast density among asymptomatic premenopausal women.

## METHODS

### Study Participants

Study subjects included the asymptomatic, pre-menopausal women between 20 to 40 years who visited the General Surgery Breast Clinic, Patel Hospital, Karachi, Pakistan between March 3 and November 10, 2015. Women eligible to be included in this study underwent measurement of serum vitamin D levels and mammography simultaneously. Women with history of breast cancer, history of breast surgery, pregnant or lactating, unknown menopausal status and taking oral contraceptives were excluded. The study was approved by Institutional review board of Ziauddin University Hospital. Written informed consent was obtained from all the participants prior to recruitment in the study, and anonymity and confidentiality of the data was maintained throughout the research.

### Serum 25 (OH) D and Calcium Analysis

The serum samples from patients were obtained from median cubital vein, and serum 25 (OH) D and serum Calcium levels were assayed via chemiluminescence immunoassay. All patients were sent to the radiology department for mammography examination on the same day after their serum samples were taken. A minimum difference of one hour was kept between serum sampling and mammographic examination.

### Mammographic Breast Density

Two standard screening views, i.e., cranial–caudal (CC) and medio-lateral oblique (MLO) of each breast from all study participants were obtained using a single manufacturer x-ray system (Fuji CR Lilium) and evaluated by a single trained radiologist. MD was assessed using automated volumetric breast density software, VolparaResearch (algorithm version 1.5.1, Volpara solutions Ltd, Wellington, NZ). The physics acquisition parameters required by the software were extracted using the FCR console and manually entered into the DICOM header of the raw (“For Processing”) images. Volpara also estimates the thickness of each tissue type by comparing each individual pixel signal to a reference signal of pure fat tissue. The different thicknesses of adipose and fibro glandular tissue in the breast were converted to volumes at each pixel, then quantified and summed across the entire breast to obtain the volume of fibro glandular tissue (cm^3^) and the total breast volume (cm^3^). The volumetric breast density (VBD) was categorized as; VG1: 0% - 4.5 %; VG2: 4.6% - 7.5%; VG3: 7.6% – 15.5% and VG4 >15.5% which corresponded to American College of Radiology BI-RADS 4th edition density categories 1-4^12^.

### Data Collection

Study subjects characteristics i.e. anthropometric measurements, age at menarche, parity, number of pregnancies, number of children, age at first and last birth, and breast feeding history were inquired and recorded on a structured proforma by a single trained physician.

### Data Analysis

Qualitative variables were presented as frequency (percentage), while Quantitative variables were described as Mean ± SD. The quantitative variables were compared across the 4 BI-RADS categories using ANOVA. The qualitative variable was compared across the 4 BI-RADS categories using Chi-square statistics. Correlation analysis was performed between percent VBD and other variables of interest. Standard of statistical significance was P value less than 0.05. All statistical analyses were performed using PASW version 21 for Windows (SPSS, Chicago, IL).

## RESULTS

In the descriptive cross-sectional survey conducted, one hundred and twenty four participants were enrolled. The response rate was over eighty percent, with hundred participants responding and gave informed consent for mammogram and laboratory testing. Among, enrolled pre-menopausal female participants, majority (78%) were married with mean (SD) age as 31.60 (5.95). The age range of study participants enrolled was 20 – 40 years. The mean (SD) for percentage Volumetric Breast Density (VBD) was 10.88 (5.77). Using the cut-off for classification of percent volumetric density, majority (46%) lied in VBD3, followed by VBD2 (26%), and VBD (21%). Only seven percent of the participants enrolled were in VGI category.

The comparison of study participant’s characteristics across the four percent volumetric breast density Glandular Volume(VG) categories is shown in Table-I. Among the characteristics compared, only age (years), weight (kg), BMI (Kg/m^2^) and parity were found significant. The mean of the anthropometric measurements (weight and BMI) were comparatively higher in VG2, followed by VG1, VG3 and least mean values in VG4. Moreover, greater proportion of study participants with parity were found in VG1 (100%), followed by VG2 (84.6%), VBD3 (67.4%) and VG4 (47.6%) with p-value = 0.013.

**Table-I:** Comparison of characteristics of the study participants across percent volumetric breast density categories.

*Participants Characteristics*	*VG1 (n =7)*	*VG2 (n = 26)*	*VG3 (n = 46)*	*VG4 (n = 21)*	*Total (n = 100)*	*P-value*
Age (years)	35.86 ± 3.72	33.62 ± 4.67	30.80 ± 6.00	29.43 ± 6.70	31.60 ± 5.95	0.015
Weight (Kg)	63.57 ± 7.18	71.32 ± 10.71	63.23 ± 10.12	53.24 ± 11.41	63.18 ± 12.01	0.001
Height(meters)	1.56 ± 0.69	1.59 ± 0.56	1.59 ± 0.57	1.55 ± 0.09	1.58 ± 0.07	0.188
Body Mass Index (Kg/m^2^)	26.24 ± 3.73	28.56 ± 4.02	25.12 ± 4.00	22.22 ± 4.92	25.46 ± 4.70	0.001
Age at Menarche (years)	13.43 ± 1.27	12.96 ± 0.92	12.83 ± 0.85	13.30 ± 1.49	13.00 ± 1.06	0.261
Parity						
Yes	7 (100)	22 (84.6)	31 (67.4)	10 (47.6)	70 (70)	0.013
No	0 (100)	4 (15.4)	15 (32.6)	11 (52.4)	30 (30)	
Number Pregnancies	3.71 ± 1.70	3.45 ± 1.74	4.00 ± 2.08	4.20 ± 3.88	3.83 ± 2.26	0.792
Number of Children	3.14 ± 1.21	3.18 ± 1.68	3.19 ± 1.64	2.44 ± 1.13	3.09 ± 1.55	0.628
Age at first birth (years)	23.57 ± 3.87	22.33 ± 4.10	20.77 ± 4.12	23.78 ± 4.27	21.96 ± 4.19	0.148
Age at last birth (years)	28.86 ± 2.54	28.44 ± 4.05	27.82 ± 4.33	31.00 ± 5.57	28.50 ± 4.25	0.371
Child Breastfed	2.86 ± 1.21	3.14 ± 1.71	3.00 ± 1.41	2.63 ± 1.06	2.98 ± 1.21	0.854
Breast feeding duration (months)	20.79 ± 8.50	18.52 ± 11.50	20.60 ± 8.24	24.00 ± 0.00	20.23 ± 9.15	0.623

Data are means ± SD or number (%). Percent Volumetric Breast Density Categories as: VG1 (0% - 4.5%), VG2 (4.6% - 7.5%), VG3 (7.6% - 15.5%) and VG4 (> 15.5%).

**Fig.1:**
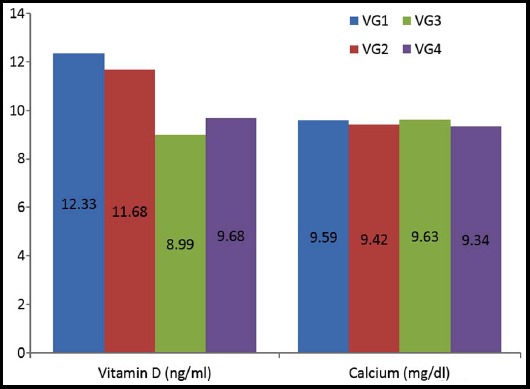
Comparison of Vitamin D(ng/ml) and calcium(mg/dl) across different volumetric breast density categories. Mean values are reported in the figure.

Importantly, no significant difference was found in mean serum vitamin D (ng/ml) and calcium levels (mg/dl) across the four VG categories (Fig.1). The mean (SD) vitamin D was found comparatively higher in VG1 (12.33 ± 8.02), followed by VG2 (11.68 ± 7.80), VG4 (9.68 ± 6.90) and least in VG3 (8.99 ± 5.74). Moreover, the serum calcium levels were highest in VG3 (9.63 ± 0.55), followed by VG1 (9.59 ± 0.53), VG2 (9.42 ± 0.67) and least in VG4 (9.34 ± 0.86).

Percent VBD were found significantly negatively correlated with age (r = - 0.365; p-value = 0.001), weight (r = - 0.575; p-value = 0.001), height (r = - 0.197; p-value = 0.049), and BMI (r = - 0.519; p-value = 0.001). Table-II. The serum Vitamin D and calcium levels were not found significantly correlated with percent volumetric breast density with p-value > 0.05.

**Table-II:** Correlation of related factors with Percent Volumetric Breast Density.

*Factors*	*Correlation coefficient*	*p-value*
Age (years)	- 0.365	0.001
Weight (Kg)	- 0.575	0.001
Height (meters)	- 0.197	0.049
Body Mass Index (Kg/m^2^)	- 0.519	0.001
Age at Menarche (years)	0.044	0.664
Number Pregnancies	0.073	0.549
Number of Children	- 0.147	0.229
Age at first birth (years)	0.003	0.982
Age at last birth (years)	0.092	0.486
Child Breastfed	- 0.122	0.345
Breast feeding duration (months)	0.181	0.169
Vitamin D (ng/ml)	- 0.149	0.139
Calcium (mg/dl)	- 0.094	0.357

## DISCUSSION

This cross-sectional study was conducted to investigate the association between serum 25 (OH) D levels and percent VBD among asymptomatic pre-menopausal women. No significant difference was found in mean serum vitamin D and calcium levels across the four volumetric breast density categories. Moreover, in correlation analysis, percent VBD was not found significantly associated with serum vitamin D and serum calcium levels. Anthropometric measurements i.e. (weight, height and BMI) and age were found negatively correlated with percent volumetric density.

Evidence from the literature is suggestive of significant association between vitamin D intake and breast density. A cross-sectional study conducted in United States among 86 premenopausal women in 2007, reported an inverse association between vitamin D intake and breast density.^10^ Another cross-sectional study conducted in 2006, among 99 premenopausal Hispanic women reported a significant inverse association between vitamin D intake and percent breast density with significant difference in mean percent breast densities as 22%, 20.35 and 9.6% were observed in the low, middle, and high dietary vitamin D intake groups.^11^ A large cross-sectional survey conducted in 2010, among multiethnic premenopausal women also reported a significant inverse association between vitamin D and calcium intake with percent breast density.^12^ The studies mentioned above significantly related vitamin D intake with breast densities.

A meta-analysis of three studies that examined the association between circulating serum 25 (OH)D levels and breast cancer risk in pre-menopausal women reported a significant inverse relationship (OR = 0.66, 95% CI = 0.50 – 0.58).^8^ Most of the cross-sectional studies showed findings consistent to our study where no significant association was found between serum 25(OH) D levels and percent breast density. A cross-sectional study of 182 multiethnic premenopausal women showed no significant association between serum 25(OH) D levels and percent breast density after adjustment for confounders (BMI, age at mammogram, Asian ethnicity, age at first birth, parity and age at menarche).^13^ A recent cross-sectional study conducted among both pre- and postmenopausal women reported no significant association between serum 25-hydroxyvitamin D and percent breast density.^14^

In the present study BMI was inversely correlated with percent breast density. Another recent study reported the similar findings where BMI was found negatively associated with breast density.^14^ A case control study reported inverse association between childhood and adult body size with breast cancer risk in premenopausal women.^15^

### Limitations of the study

Firstly, being the cross-sectional study it was not possible to establish a causal relationship between serum 25 (OH) D levels and breast density. Secondly, there may be selection bias in recruitment of study participants. Thirdly, most of the study subjects had vitamin D deficiency with only two patients having sufficient vitamin D levels. Finally, as breast density varies with the menstrual cycle in the premenopausal women, it was not possible to perform mammography of all enrolled patients at follicular phase.

## CONCLUSION

Our findings suggest that no significant association exist between serum 25 (OH) D levels and breast density but significant association existed between serum vitamin D and body mass index. However, large prospective multicentre studies are desirable to be conducted in future to confirm our suggestive findings and identify a possible causal relationship.
